# Supplementary material for: [89Zr]-Atezolizumab-PET Imaging Reveals Longitudinal Alterations in PDL1 during Therapy in TNBC Preclinical Models
Source: Cancers (Basel). 2023 May 11;15(10):2708. doi: 10.3390/cancers15102708 (PMC10216761; doi:10.3390/cancers15102708)
Supplement: Supplementary file 1 [file cancers-15-02708-s001.zip › cancers-2278238-supplementary-figures.pdf]

cancers

MDPI

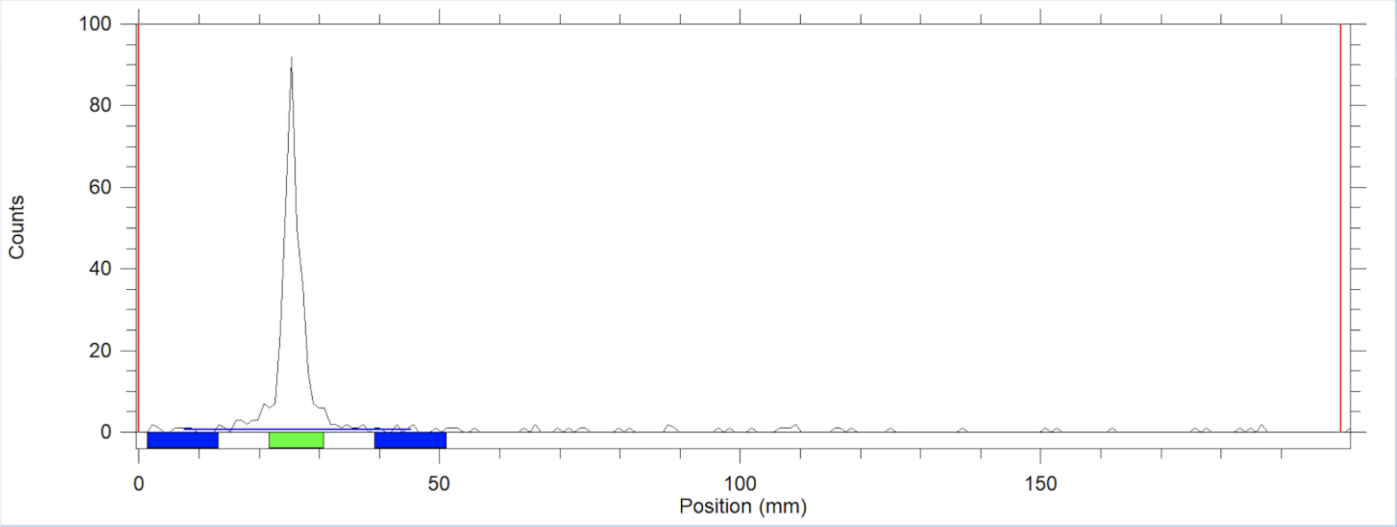

Figure S1. iTLC assessment of [^{89}Zr]-Atezolizumab demonstrates high purity and radiolabeling efficacy (mobile phase: DTPA).

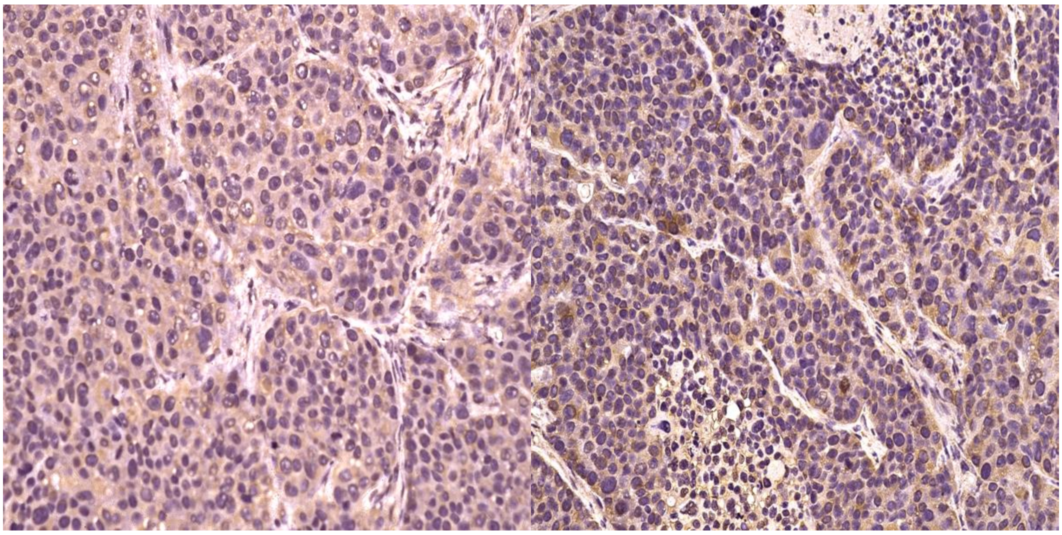

Figure S2. PD-L1 immunohistochemistry of BCM3936 demonstrates heterogeneity within cancerous regions of TNBC.
