# Supplementary material for: [89Zr]-Atezolizumab-PET Imaging Reveals Longitudinal Alterations in PDL1 during Therapy in TNBC Preclinical Models
Source: Cancers (Basel). 2023 May 11;15(10):2708. doi: 10.3390/cancers15102708 (PMC10216761; doi:10.3390/cancers15102708)
Supplement: Supplementary file 1 [file cancers-15-02708-s001.zip › cancers-2278238-supplementary-methods.pdf]

Reagents and materials

[^{89}Zr]oxalate was produced at the Cyclotron Facility at University of Alabama at Birmingham (UAB) from the ^{89}Y(p,n)^{89}Zr reaction using ^{89}Y sputtered targets as described previously (*25*). Atezolizumab (Tecentriq®) was purchased from Genentech (San Francisco, CA). Desferrioxamine-p-benzyl-isothiocyanate (DFO-Bz-NCS) was purchased from Macrocyclics (Dallas, TX). All other chemicals were purchased from Fisher Scientific (Hampton, NH) except where otherwise stated.

Tissue Culture

Breast cancer cell lines MDA-MB-231 and HCC38 were purchased from American Type Culture Collection (ATCC) and cultivated in Dulbecco's Modified Eagle Medium (DMEM) and Roswell Park Memorial Institute 1640 (RPMI) respectively with 10% FBS and gentamycin (50 mg/mL) in a humidified incubator with 5% CO_2 at 37 ˚C. All other reagents for cell culture were purchased from Gibco® Life Technologies (Grand Island, NY).

Animal Models

Evaluation of in vivo specificity with PET imaging and biodistribution
Five-week-old female Balb/c NU/NU nude mice (Charles River; Wilmington, MA) were subcutaneously implanted with $1x10^7$ MDA-MB-231 cells on the right shoulder and the tumors were allowed to grow for 3 weeks.

Quantitative analysis of [^{89}Zr]Atezolizumab tumor uptake as a tool to measure PD-L1 expression
Five-week old female NSG mice were purchased from Jackson Laboratories (Bar Harbor, ME) and transplanted with TNBC PDX BCM 3936 (*31*) (Patient-derived Xenograft and Advanced In Vivo Models Core; Baylor College of Medicine; Houston, TX) tumor into the 3rd mammary fat pad and the tumors were allowed to grow for 4 weeks. When the tumors were approximately 300 mm^3 (298.9 $mm^3 \pm$ 70.3 mm^3), mice were enrolled into experiment.

Evaluation of [^{89}Zr]Atezolizumab-PET imaging to assess changes in PD-L1 expression following cytotoxic treatment in TNBC preclinical PDX models.
Five-week old female NSG mice were transplanted with TNBC PDX BCM 3936 as previously described and allowed grow to approximately 300 mm^3 in size (308.9 $mm^3 \pm$ 113.3 mm^3). The *niraparib group* received 50 mg/kg of niraparib on day 0, 1, 2, and 3 via oral gavage; *paclitaxel group* received 10 mg/kg of paclitaxel on day 0, and 3 via IP injection; *radiation group*, received 4 Gy of localized X-ray radiation (tumor) on day 0, 1, 2 and 3; and the *control group* received 100 μL of saline IP on day 0, 1, and 3.

Immunohistochemistry (IHC) and quantification of PD-L1 expression

Immediately following imaging, BCM 3936 PDX tumors were collected and fixed in 10% formalin followed by 70% ethanol for sectioning. Tumor sections were stained with a 1:100 dilution of anti-human PD L1 (ab210931, Abcam, Cambridge, MA) overnight, followed by goat anti-mouse secondary antibody (VC002, R&D Systems, Minneapolis, MN) for 3 hours. Positive staining was visualized with HRP DAB substrate kit (SK-4100, Vector Laboratories, Burlingame, CA) and nuclear counterstained with hematoxylin.

Custom MATLAB code were developed to automatically quantify PD-L1+ IHC signaling and necrosis (H&E) in whole cross-sectional tumor slices. Whole tumor sections were imaged (EVOS M7000 Imaging, Thermofisher; Waltham, MA). Segmentation of diaminobenzideine and hematoxylin was accomplished by adapting the scikit-learn color deconvolution (*33*). Afterwards, a binary mask was formed by applying a locally adaptive threshold to each image. To minimize false positive signal, nonconnected areas of less than 100 pixels were omitted. Positive IHC percentage was calculated as the fraction of the positive pixel count of total pixels within the segmented tumor section.
